# Supplementary material for: Colorimetric Assaying of Exosomal Metabolic Biomarkers
Source: Molecules. 2023 Feb 16;28(4):1909. doi: 10.3390/molecules28041909 (PMC9962048; doi:10.3390/molecules28041909)
Supplement: Supplementary file 1 [file molecules-28-01909-s001.zip › molecules-2166733-supplementary.pdf]

molecules

Supplementary Information

Article

Colorimetric Assaying of Exosomal Metabolic Biomarkers

Evelias Yan [1,2,†], Garima Goyal [1,2,3,†], Umit Hakan Yildiz [4], Bernhard O. Boehm [5] and Alagappan Palaniappan [1,2,*]

[1] Center for Biomimetic Sensor Science, Nanyang Technological University, Singapore 637553, Singapore; evelias.yan@ntu.edu.sg (E.Y.); garima170892@gmail.com (G.G.)
[2] School of Materials Science and Engineering, Nanyang Technological University, Singapore 639798, Singapore
[3] Interdisciplinary Graduate School, Nanyang Technological University, Singapore 637335, Singapore
[4] Department of Chemistry, Izmir Institute of Technology, 35430 Izmir, Turkey; hakanyildiz@iyte.edu.tr
[5] Lee Kong Chian School of Medicine, Nanyang Technological University, Singapore 308232, Singapore; bernhard.boehm@ntu.edu.sg
* Correspondence: alps@ntu.edu.sg;
† These authors contributed equally to this work.

Citation: E. Yan; Goyal, G.; Yildiz, U.H.; Boehm, B.O.; Palaniappan, A. Colorimetric Assaying of Exosomal Metabolic Biomarkers. *Molecules* **2023**, *28*, 1909. https://doi.org/10.3390/molecules28041909

Academic Editor: Atul N. Parikh

Received: 31 December 2022
Revised: 10 February 2023
Accepted: 15 February 2023
Published: 16 February 2023

Copyright: © 2023 by the authors. Licensee MDPI, Basel, Switzerland. This article is an open access article distributed under the terms and conditions of the Creative Commons Attribution (CC BY) license (http://creativecommons.org/licenses/by/4.0/).

Characterization of exosomes

Cryo-electron microscopy were used to characterize the morphology and size of the isolated exosomes, before and after lysing with 0.1% Triton X-100. As shown in Figure 2a, the digital image from the exosome sample before lysis shows a spherical vesicle of size approximately 90 nm. This indicates that the vesicles in the exosome sample before lysis remains intact. Conversely, in Figure 2b and Figure S1, the digital images from the exosome after lysis show a random size distribution of particles. This shows that the vesicles have been ruptured after lysing with 0.1% Triton X-100.

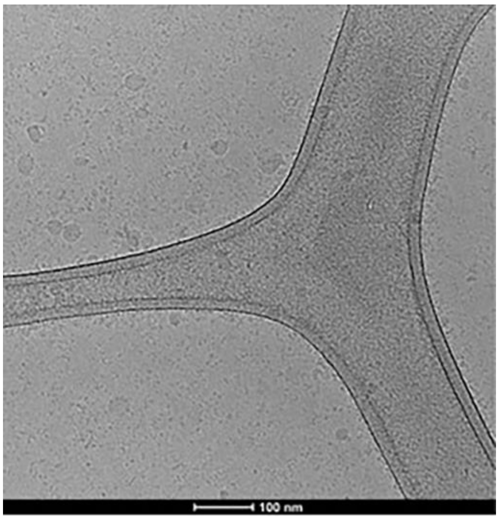

Figure S1. Electron microscopy image of exosome after lysis

HBV-DNA Assay

For the HBV-DNA assay, 10 µL of various concentrations of HBV-DNA spiked exosomes samples were added to 10 µL of 5 µM complementary PNA 2 and also to 10 µL of PBS (without PNA), incubated for 30 min, and followed by the addition of 30 µL of 60 uM PT-T20 solution. Figure S2a-d, PT-T20-PNA2 (PNA sequence complementary to HBV-DNA) was explored for the detection of exosomal HBV-DNA. Upon colorimetric, ΔE and

fluorometric responses analysis, a colorimetric LOD of 0.12 fM/exosome and a fluorometric LOD of 0.04 fM/exosome was evaluated.

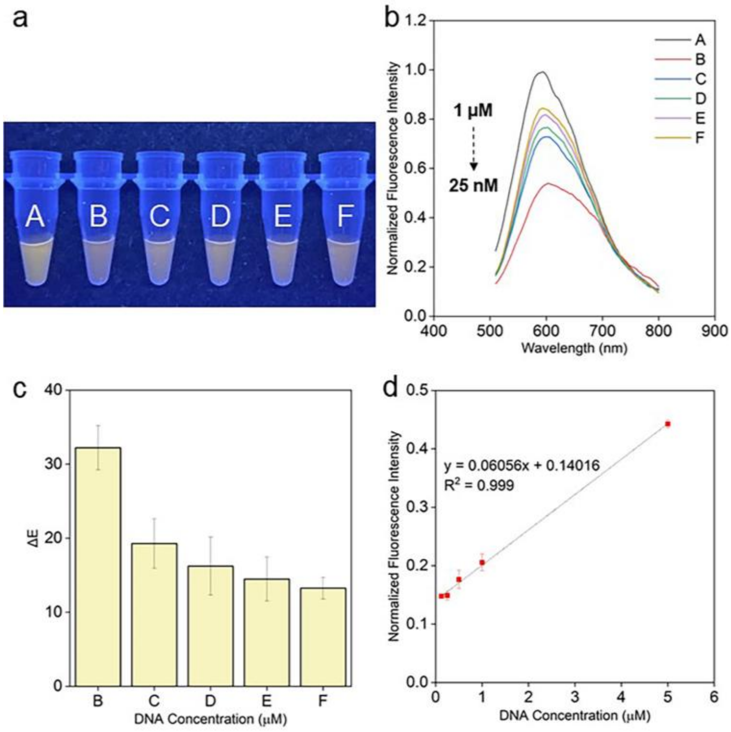

Figure S2. HBV DNA Assay (**a-d**): (**a**) digital images of vials containing (A) PT−T20, PNA 2, and 0.2 μM HBV DNA (control), and (B-F) PT-T20 with 1 μM to 25 nM HBV DNA (vials: B, 1 μM; C, 200 nM; D, 100 nM; E, 50 nM; F, 25 nM) and, (**b**) ΔE values calculated according to vial A, (**c**) their corresponding fluorescence spectra and, (**d**) Calibration curve.

AGE Assay

For the protein (AGE) assay, 15 μL of various concentrations of AGE spiked diluted 10 times exosomes samples were added to 5 μL of 1 μM aptamer (Apt 1) and incubated for 30 min. After that, 30 μL of 120 μM PT-T20 solution was added.

In addition to nucleic acid assaying, the proposed assay was utilized to detect exosomal AGE with the use of an aptamer (Apt) as the recognition element, as shown in Figure S3a-d. The colorimetric responses, ΔE analysis, fluorescence spectra and calibration curve were established. Figure S3a shows the colorimetric responses of various concentrations of AGE (vials: B, 974 nM; C, 877 nM; D, 779 nM; E, 682 nM; F, 584 nM). With Apt 1 (vial A, control), PT-T20-Apt shows a purple colour, indicating a fluorescence quenching. As the concentration of AGE decreases (from 974 nM (vial B) to 584 nM (vial F)), the colour changes from orange to purple as shown in Figure S3a. The ΔE value was then evaluated as shown in Figure S3b, which shows that the PT-T20-Apt and PT-T20-Apt-AGE can be visually distinguished. The colorimetric limit of detection was calculated based on the 3σ/S approach, which yields a LOD of ~ 0.63 fM/exosome. Furthermore, the fluorescence spectra show that as the concentration of AGE decreases, the fluorescence intensity decreases, as illustrated in Figure S3c. The maxima of each AGE concentration were plotted as a linear function, that shows the fluorescence intensity decreases gradually from 974 nM to 584 nM of AGE, as shown in Figure S3d, yielding a LOD of ~ 0.34 fM/exosome using the 3σ/S approach. Thus, the proposed approach illustrates that colorimetric detection of the different exosomal biomarkers is feasible.

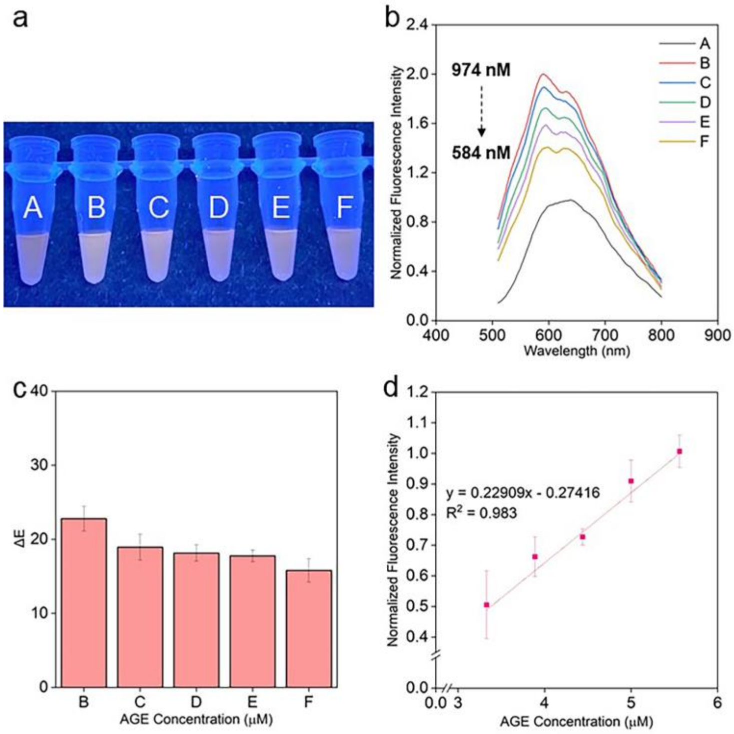

Figure S3. AGE Assay (**a-d**): (**a**) digital images of vials containing (A) PT−T20 and Apt 1 (control), and (B-F) PT-T20 and Apt 1 with 974 nM to 584 nM AGE (vials: B, 974 nM; C, 877 nM; D, 779 nM; E, 682 nM; F, 584 nM) and, (**b**) ΔE values calculated according to vial A, (**c**) their corresponding fluorescence spectra and, (**d**) Calibration curve.

LODs Calculation

For Figure 6d, the fluorescence maxima of each concentration of mir21 were then plotted against the various concentrations of mir21 and it shows that the fluorescence intensity increases gradually from 500 nM to 4 μM of mir21. The maxima values for each concentration of mir21, averaged for three individual experiments (n = 3) and plotted as a linear function of 4 μM to 250 nM exosomal mir21 concentration, yielded a LOD of 1.2 fM/exosome, using the 3σ/S approach.

The LOD is obtained using the following calculations:

Linear equation of the plot: y=0.1264x+ 0.0594 with a standard deviation of 0.025 gives lowest mir21 concentration to be distinguishably detected to be 600 nM in 50 μL of extracted exosome solution

Exosome sample volume: 5 mL

1 x 10^{12} - 3 x 10^{12} vesicles in 1 mL serum (of which 5-10% are exosomes, the rest mostly lipid vesicles).

10^{12} vesicles/mL of serum

0.5 x 10^{11} exosomes/mL of serum

Take 10 μL of 0.5 x 10^{11} exosomes/mL of serum

In final 50 μL → 1 x 10^{10} exosome/mL = 5 x 10^{8} exosomes

600 nM / 5 x 10^{8} exosomes = 1.2 x 10^{-6} nM/exosome

1.2 fM / exosome
